# Psychological capacity profiles of different age groups and gender in a national representative sample

**DOI:** 10.1002/pchj.795

**Published:** 2024-08-26

**Authors:** Beate Muschalla

**Affiliations:** ^1^ Institute of Psychology Technische Universität Braunschweig Braunschweig Germany

**Keywords:** age groups, coping, gender, life span, psychological capacities, soft skills

## Abstract

Beyond the specific cognitive capacities like numerical or verbal intelligence and cognitive speed, the so‐called soft skills, namely, psychological capacities, have become highly important in modern life. This is the first representative study on the distribution of work‐relevant psychological capacities in the general population. We investigate capacities in different age groups, gender, and their relation with basic sociodemographics. A representative sample of 2531 people aged 14–95 years was investigated concerning work‐relevant psychological capacities with the mini self‐rating for psychological activities and participation (Mini‐ICF‐APP‐S). The strongest capacities in young people were mobility, flexibility, proactivity, contact to thirds, and group interaction. Other capacities were stronger in midlife (30–59 years), such as adjustment to rules and routines, planning and structuring, decision making and judgement, application of competence and knowledge, assertiveness, dyadic relationships, endurance, and self‐care. Women reported better dyadic relationship capacities, and men felt more assertive. The study provides, for the first time, representative data on a broad range of psychological capacities according to an internationally validated capacity concept. Good psychological capacities occur not primarily in youth, but especially in midlife and older age. Regarding demographic change, this implies older people are highly competent in the working world.

## INTRODUCTION

### Work‐relevant human (cognitive) capacities over the life span

Modern working life demands psychological capacities from the employees. There is a long research tradition on human capacities and skills, including cognitive skills. Theories, as well as empirical research, shed light on cognitive (e.g., Klindt et al., 2017), motor (Schirinzi et al., 2020), moral (Thalmeyer et al., 2019), social and emotional (Blanke et al., 2016) development of skills and capacities. They also describe complex interactions of different capacity domains (Thalmeyer et al., 2019).

It was found that cognitive and social capacity development must be regarded over the whole life span, not only from childhood to adult status (Janacsek et al., 2012; Lukács & Kemény, 2015). Different aspects of cognitive and social skills must be regarded differently, and they do not develop linearly and similarly over the life span (Ardila et al., 2000). For example, children are especially good in procedural learning, in both general and statistical learning (Juhasz et al., 2019). In young age groups, the value of good initial educational and learning conditions is setting the course (Ardila et al., 2000; Lövdén et al., 2020). The maturation of executive functions plays a role for establishing distinct cognitive architectures and mechanism of theory of mind, namely, reading other peoples' emotional and motivational states and thoughts (Klindt et al., 2017). Educational attainment even impacts on late‐life cognitive functions. Education contributes to individual differences in cognitive skills that emerge in early adulthood but persist into older age (Lövdén et al., 2020). There are also genetic influences on cognitive and functional capacities (especially fluid intelligence functions such as speed; Proust‐Lima et al., 2008), or numeracy skills (Fastame et al., 2019), which become significant in older ages (Arden et al., 2016; Ardila et al., 2000; Schupf et al., 2004).

In sum, research has until now focused on very specific (cognitive) capacities. By contrast, there is until now not much known about the distribution of psychological capacities in the general population, covering all capacity domains and age groups.

### The relevance of psychological capacities

Beyond the specific cognitive capacities like numerical or verbal intelligence functions and cognitive speed, the so called soft skills, that is, psychological capacities, become more and more important in education and work settings (Geisinger, 2016; OECD, 2019). “Soft skills” include capacities with a broader range. They include not only cognitive but also action‐based, social capacities: flexibility, competence, decision making and judgment, and social skills like contacting others, group and teamwork capacity (WHO, 2001). Beyond specific aspects of numerical or verbal intelligence or cognitive speed, these capacities are generally important in school, in, and in social life (Geisinger, 2016; OECD, 2019) in all age groups (Blanke et al., 2016).

The importance of such psychological capacities, or “soft skills,” is especially seen in the working world, in which service jobs are dominating, employees are more and more narrowly (computer‐)monitored concerning their achievements, and work outcomes are compared due to competition. Sick leave may occur when there is a misfit between psychological work demands and person capacities (Muschalla, 2016).

There are until now hardly data on the distribution of soft skills, namely, psychological capacities, in the general population. To know about the people's psychological capacity levels and profiles in different age groups or gender is useful in order to prevent potential problems with capacity‐demand‐misfit in education or professional context. In times of demographic change, older age groups become even more relevant to stay in active and professional life. They apply their knowledge and skills and teach the younger. The need to strengthen intergenerational exchange and coworking of people from different age groups are important topics in work and organizational research (Santos & Cox, 2000). Also for this purpose it is important to have some knowledge about the psychological capacity level and profiles of different age groups.

### Psychological capacities according to the Mini‐ICF‐APP (mini rating for psychological activities and participation)

This present study is the worldwide first study to assess the broad set of psychological capacities in a large national representative cohort (Linden et al., 2009). The hereby used *concept of psychological capacities* is internationally validated and applied. This capacity concept covers psychological core capacities which are commonly necessary in school, professional, and general social life (mini rating for psychological activities and participation, Mini‐ICF‐APP; AWMF, 2019; Balestrieri et al., 2013; DRV, 2012; SGPP, 2012; Linden et al., 2009; Linden, Keller, et al., 2018; Molodynski et al., 2013; Muschalla, 2018; Pinna et al., 2015). The Mini‐ICF‐APP capacity concept is based on a capacity definition introduced in the International Classification of Functioning Disability and Health (ICF) by the World Health Organization (WHO, 2001). The capacity construct reflects the environmentally‐adjusted ability of the individual to carry out certain activities in a specified domain. The ICF‐based Mini‐ICF‐APP capacity assessment comprises 13 psychological capacity dimensions (Linden et al., 2009; Linden, Keller, et al., 2018):

- Cognitive and action‐oriented capacities: adherence to regulations, planning and structuring of tasks, flexibility and ability to adapt to changes, competency and application of knowledge, ability to make decisions and judgments, proactivity and spontaneous activity, endurance and perseverance
- Social capacities: assertiveness, contact with others and small talk, group interaction, dyadic or close relations
- Basic capacities: mobility, self‐care

Each *capacity* may include *several activities*. For example, the capacity “adherence to regulations” includes activities such as being on time to meetings and dates, working on a work piece or according to specific rules, obeying rules in traffic, or work routines, and so on.

Capacities can be assessed by observer rating or self‐rating. It could be argued that self‐ratings may have some biases and differ from observer ratings, which indicates reduced validity of self‐rating. However, empirical data show that self‐ and observer‐rating are often corresponding: Clinical studies found the ranking and type of impairments reported similarly by observing therapist and self‐rating patient. The only difference is that patients usually report their capacities weaker than therapists who observe these capacities (e.g., Linden, Deck, & Muschalla, 2018). Another indicator for the validity of self‐rated capacities is that there are differences between persons from the general population and psychotherapy patients: patients rate themselves worse than the general population persons (Linden, Keller, et al., 2018). This makes sense, as patients are normally more impaired than healthy persons. Thus, capacity profiles and interpersonal differences in capacity levels seem to be measureable by self‐rating.

Psychological capacities appear in different quality or strength over the life span, in different age groups, or in people with mental disorders and in the general population (Linden, Keller, et al., 2018). Capacities in which people perceived themselves generally as most competent were mobility, dyadic relationships, group interaction, competency, decision making, adherence to rules and regulations. Endurance and assertiveness were rated more restrained not only by patients, but also by the general population participants (Linden, Keller, et al., 2018).

Representative data on work‐ and life‐relevant psychological capacities in the general population have not been reported yet. To fill in this gap, the present representative investigation will focus on the following three main objectives:

1. Which profiles of psychological capacities can be found in different age groups? Are there differences between capacity levels in different age groups?
2. (To which degree) Is the overall perceived capacity level associated with basic sociodemographic characteristics?
3. Are there differences in self‐perceived capacity levels in men and women?

## MATERIALS AND METHODS

A representative survey has been done throughout Germany in 2019. The survey has been conducted by a professional organization for representative survey (USUMA, 2019). The project with the title “Psychological capacities in a representative study” was financially supported by the German Federal Pension Agency, Grant Number 0421/40‐64‐50‐01. The ethical approval has been obtained from the Faculty of Liefe Science, Technische Universität Braunschweig, number D‐2019‐03.

### Participants

Full data for analysis were available from 2531 participants, of which 53.3% were female. Average age of all participants was 48.4 (*SD* = 17.8); 43.7% were married, 37.5% were Protestants, 30.2% Catholics, 2.3% Muslims, 3.3% other religions, and 26.6% without religious denomination; 27.3% had finished eight years at school, 44.5% left school after 10 years, 12.8% had A‐levels/high school degree with 12–13 school years, 2.9% were still at school; 9.9% had an additional college or university degree. Two thousand and thirty participants were in the working age, namely, between 18 and 67 years of age.

### Materials and procedure

First, basic socio‐demographic and profession‐related questions were asked in the interview. Then, participants filled in the mini self‐rating for psychological activities and participation (Mini‐ICF‐APP‐S; Linden, Keller, et al., 2018).

Mini‐ICF‐APP‐S (mini self‐rating for psychological activities and participation; Linden, Keller, et al., 2018). It is often suggested that the assessment of capacities and capacity limitations should be based on expert rating. However, given that self‐perceived work ability is a strong predictor for future real work ability (de Vries et al., 2018), important information can also be obtained from capacity self‐ratings. A self‐rated capacity profile reflects the self‐image of a person, may give a hint towards possible aggravation tendencies, and provide information for further therapy planning, capacity training, or work adjustment.

The Mini‐ICF‐APP‐S is a self‐rating on psychological capacities (Linden, Keller, et al., 2018). It covers the same 13 capacity dimensions (Table 2) as the original internationally validated and established *observer rating Mini‐ICF‐APP* (Linden et al., 2009; AWMF, 2019; Balestrieri et al., 2013; Molodynski et al., 2013; Pinna et al., 2015). Similar to the observer rating, the Mini‐ICF‐APP‐S self‐rating includes 13 items which each represents one capacity dimension. Descriptions of each capacity dimension are given. The rating points are described at a behavioral level, namely, the degree to which the person can (or has problems to) act out capacity‐related activities. The self‐rating allows a bipolar rating from “*7* = *this is clearly a strength of mine*” to “*4* = *this is somehow possible*”, “*3* = *this does not always work*” to “*0* = *I am fully unfit to do this*.”

The strength or weakness of a capacity is always judged in relation to contextual demands. The self‐rating Mini‐ICF‐APP‐S does not give a specific reference context for the qualification of the self‐rated psychological capacities. Therefore, the subjective norms or demands underlying the subjective ratings may vary, or be specific (e.g., In my working team, I am the most enduring person), or broad (e.g., Comparing myself with others in my work or family or freetime life, I need more help in planning and structuring my activities). Despite this little vagueness, the bipolar rating with the eight differently described scale points makes it possible to describe psychological capacities as a relative subjective strength or weakness.

Each capacity item can be interpreted for itself, because it reflects one specific capacity dimension out of 13. If a global score is calculated, namely, the mean score over all 13 capacity items, this mean score should be interpreted as the level of overall capacity level.

The Mini‐ICF‐APP‐S self‐rating has been validated in a sample of patients with mental disorders, and a general population sample (Linden, Keller, et al., 2018). The original Mini‐ICF‐APP has been validated with an established structured *Groningen Social Disability Interview* (Linden et al., 2009; Wiersma et al., 1990). The capacity assessment has good inter‐rater reliabilities between *r* = 0.70–0.90, and has been evaluated in translated in several languages and cultural contexts (AWMF, 2019; Balestrieri et al., 2013; Molodynski et al., 2013; Pinna et al., 2015).

### Statistical analysis

Data have been analyzed with SPSS. Descriptive statistics, and group comparisons by analysis of variance (T‐test or ANOVA, with Bonferroni correction) or Chi^2^‐test have been calculated.

## RESULTS

In all capacity dimensions, more than 50% of participants say they can do this at least “quite good,” and up to 40%–50% say that capacities are “clearly a strength” of theirs, or that they can do this “better than most others.”

### Capacity profiles in different age groups

Different age groups have significantly different self‐perception of their psychological capacities (Table 1). Very *young people* (14–19 years) perceive themselves able to adjust to rules and routine “somehow,” whereas older age groups—from second to seventh life decades—are to a similar degree convinced that they “can do this well.” *Older age groups* up from the sixth life decade are less convinced about their mobility capacities: they say this is “somehow” possible, whereas teenagers and people in their twenties believe that they are “better than many others.” However, people in their seventies see themselves still able to adjust to rules and routines, care for themselves, and interact in dyadic relationships, similar to the youngsters and midagers. *Midagers* are especially convinced about their endurance in their third and fourth decade of life, and do positively value their competence up from the twenties to the forties.

**TABLE 1:** Self‐perceived psychological capacities according to mini self‐rating for psychological activities and participation (Mini‐ICF‐APP‐S) in different age groups from a national representative sample (*N* = 2531).

Capacities	Age 1 14–19 (*n* = 141)	Age 2 20–29 (*n* = 319)	Age 3 30–39 (*n* = 386)	Age 4 40–49 (*n* = 416)	Age 5 50–59 (*n* = 511)	Age 6 60–69 (*n* = 432)	Age 7 70–79 (*n* = 241)	Age 8 80–95 (*n* = 85)	Significant differences between age groups (ANOVA with Bonferroni correction) *p*‐value at .05
Regulations	4.65 (1.34) 21.5%	5.20 (1.24) 36.4%	5.16 (1.20) 34.7%	5.13 (1.15) 33.1%	5.24 (1.16) 34.6%	5.14 (1.17) 31.0%	5.05 (1.07) 24.7%	4.86 (1.19) 23.6%	1vs2/3/4/5/6/7
Planning	4.53 (1.32) 19.3%	5.01 (1.28) 31.7%	5.12 (1.22) 34.7%	5.19 (1.20) 37.4%	5.10 (1.24) 32.5%	5.00 (1.21) 29.5%	4.86 (1.09) 22.7%	4.47 (1.25) 18.9%	1vs2/3/4/5/6
									2vs1/8
									3vs1/8
									4vs1/7/8
									5vs1/8
									6vs1/8
									7vs4
									8vs 2/3/4/5/6
Flexibility	4.57 (1.28) 20.0%	5.11 (1.14) 36%	4.99 (1.12) 29.1%	4.92 (1.16) 28.3%	4.80 (1.17) 23.0%	4.58 (1.17) 16.6%	4.29 (1.28) 12.7%	3.70 (1.40) 30.1%	1vs2/3/4/8
									2vs1/5/6/7/8
									3vs1/6/7/8
									4vs6/7/8
									5vs2/7/8
									6vs2/3/4/7/8
									7vs2/3/4/5/6/8
									8vs1/2/3/4/5/6/7
Competence	4.67 (1.11) 17.2%	5.21 (1.08) 33.1%	5.18 (1.08) 33.9%	5.15 (1.11) 34.5%	5.13 (1.15) 31.9%	4.91 (1.14) 24.9%	4.70 (1.15) 19.1%	4.30 (1.33) 12.2%	1vs2/3/4/5
									2vs1/6/7/8
									3vs1/6/7/8
									4vs1/6/7/8
									5vs1/7/8
									6vs2/3/4/8
									7vs2/3/4/5
									8vs2/3/4/5/6
Decision	4.60 (1.12) 16.4%	4.15 (1.04) 31.5%	5.12 (1.11) 32.1%	5.10 (1.08) 30.4%	5.07 (1.14) 30.7%	4.99 (1.06) 28.3%	4.88 (1.06) 22.4%	4.65 (1.12) 17.6%	1vs2/3/4/5/6
									2vs1/8
									3vs1/8
									4vs1/8
									5vs1/8
									6vs1
									8vs2/3/4/5
Proactivity	4.63 (1.30) 21.4%	5.01 (1.21) 32.8%	4.97 (1.30) 30.4%	4.89 (1.26) 28.0%	4.84 (1.26) 25.3%	4.68 (1.26) 19.5%	4.29 (1.45) 17.3%	4.11 (1.40) 11.8%	2vs6/7/8
									3vs6/7/8
									4vs7/8
									5vs7/8
									6vs7/8
									7vs2/3/4/5/6
									8vs2/3/4/5/6
Perseverance	4.50 (1.41) 20.0%	4.94 (1.22) 30.0%	4.97 (1.27) 32.7%	5.00 (1.25) 31.0%	4.93 (1.26) 28.6%	4.86 (1.21) 25.3%	4.54 (1.28) 19.8%	4.22 (1.39) 14.1%	1vs2/3/4/5
									2vs1/7/8
									3vs1/7/8
									4vs1/7/8
									5vs1/7/8
									6vs7/8
									7vs2/3/4/5/6
									8vs2/3/4/5/6
Self‐assertion	4.81 (1.23) 35.7%	4.94 (1.35) 33.8%	4.90 (1.27) 30.5%	4.84 (1.29) 27.4%	4.83 (1.27) 25.8%	4.82 (1.21) 23.6%	4.63 (1.25) 21.0%	4.40 (1.42) 17.8%	2vs8
									3vs8
									8vs2/3
Contact	4.85 (1.41) 27.0%	5.11 (1.40) 37.6%	5.06 (1.29) 33.5%	5.07 (1.19) 30.8%	4.86 (1.30) 28.2%	4.82 (1.31) 26.4%	4.75 (1.36) 26.8%	4.56 (1.12) 12.9%	2vs7/8
									3vs8
									4vs8
									7vs2
									8vs2/3/4
Group	4.93 (1.17) 27.6%	5.16 (1.15) 35.5%	4.98 (1.26) 26.7%	4.97 (1.19) 26.9%	4.89 (1.16) 25.2%	4.73 (1.15) 19.0%	4.58 (1.31) 19.2%	4.45 (1.24) 13.1%	2vs5/6/7/8
									3vs7/8
									4vs7/8
									5vs2/7/8
									6vs2
									7vs2/3/4/5
									8vs2/3/4/5
Dyadic relations	4.76 (1.16) 19.8%	5.09 (1.30) 37.0%	5.03 (1.32) 32.7%	5.06 (1.21) 30.6%	4.90 (1.24) 27.5%	4.78 (1.14) 19.9%	4.73 (1.14) 16.2%	4.49 (1.26) 14.1%	2vs6/7/8
									3vs8
									4vs6/7/8
									6vs2/4
									7vs2/4
									8vs2/3/4
Self‐care	4.89 (1.24) 29%	5.19 (1.29) 38.9%	5.12 (1.26) 36.3%	5.20 (1.19) 36.3%	5.04 (1.29) 31.7%	5.06 (1.15)	4.96 (1.15) 25.8%	4.67 (1.19) 20.0%	8vs2/4
Mobility	5.51 (1.14) 49.6%	5.74 (1.07) 56.4%	5.47 (1.13) 43.8%	5.43 (1.07) 43.7%	5.23 (1.14) 34.5%	4.99 (1.12) 25.5%	4.51 (1.37) 18.7%	3.84 (1.59) 10.6%	1vs6/7/8
									2vs3/4/5/6/7/8
									3vs2/6/7/8
									4vs2/6/7/8
									5vs2/6/7/8
									6vs1/2/3/4/5/7/8
									7vs1/2/3/4/5/6/8
									8vs1/2/3/4/5/6/7

*Note*: Each capacity dimension is rated from *7 = This is clearly a strength of mine* to *0 = I am fully unfit to do this*. Means (standard deviation) are reported and percentage of participants who say that this capacity is clearly a strength of them or that they are better than most others in doing this (rating 6–7 on the respective capacity).

These differences between age groups show that *some capacities reach their peak in the earlier life decades*: These capacities—which are most prominent with high degrees (rating 6–7) in the teens and twenties—are mobility, flexibility, proactivity, and the interactional capacities contact to thirds and group interaction.

*Other capacities rather develop over the life span*: They are rated stronger in midlife (30–59 years), such as adjustment to rules and routines, planning and structuring, decision making and judgement, application of competence and knowledge, assertiveness, dyadic relationships, endurance, and self‐care.

### Capacity level and sociodemographic characteristics

There are some, but rather small correlations of the general perceived capacity level and basic sociodemographics (Table 2).

**TABLE 2:** Spearman correlations of overall level of psychological capacities according to mini self‐rating for psychological activities and participation (Mini‐ICF‐APP‐S) (mean score) with sociodemographics.

	Age 1 14–19 (*n* = 141)	Age 2 20–29 (*n* = 319)	Age 3 30–39 (*n* = 386)	Age 4 40–49 (*n* = 416)	Age 5 50–59 (*n* = 511)	Age 6 60–69 (*n* = 432)	Age 7 70–79 (*n* = 241)	Age 8 80–95 (*n* = 85)	All (*N* = 2531)
Sex 1: male 2: female	.105	.068	−.011	−.047	−.047	−.019	−.135*	−.145	−.029
Partnership 1: yes 2: no	−.274**	.005	−.014	−.173*	−.133	−.025	−.228**	−.145	−.151**
Monthly income	.246*	.189**	.254**	.270**	.252**	.284**	.251**	.366**	.259**
Times unemployed	−.052	−.139*	−.234**	−.261**	−.269**	−.280**	−.043	−.161	−.187**

*Note*: Mini‐ICF‐APP‐S mean scores from 7 = *This is clearly a strength of mine* to *0* = *I am fully unfit to do this*. *N* = 2531 people of a national representative sample. Level of significance: **p < .01; *p < .05, 2‐sided.

The overall capacity level seems to be systematically higher in male persons in older age, in the seventh life decade. In all other age groups the selfrated capacity level is independent from sex.

Younger (14–19 years) and older (70–79 years) people perceive a higher capacity level in case they are in a partnership. In most other (midlife) age groups partnership is not associated with higher or lower capacity level.

*Economic aspects* are more consistently associated with capacity level: Higher monthly income is associated with higher capacity level. This can be seen consistently in all age groups. The more often people in working age (20–69 years) have been unemployed, the lower they perceive their capacity level.

### Capacity differences in men and women

About 25%–30% of the representative sample report good capacities (Tables 1 and 3). The overall capacity level is not significantly associated with sex in most age groups (Table 2). Men and women are overall similarly convinced about the level of their capacities (Table 3).

**TABLE 3:** Self‐perceived psychological capacities according to mini self‐rating for psychological activities and participation (Mini‐ICF‐APP‐S) in men and women (*N* = 2531).

Capacities	Men (*N* = 1181)	Women (*N* = 1350)	All (*N* = 2531)	Significant differences between women and men *p*‐value Cohen's *d*
Regulations	5.06 (1.23)	5.18 (1.14)	5.12 (1.19)	*p* = .018
	31.7%	32.2%	31.9%	*d* = 0.094
Planning	4.99 (1.28)	5.03 (1.20)	5.01 (1.24)	*p* = .490
	31.9%	29.9%	30.9%	*d* = 0.028
Flexibility	4.81 (1.26)	4.70 (1.19)	4.75 (1.22)	*p* = .028
	27.1%	20.6%	23.7%	*d* = −0.088
Competence	5.14 (1.13)	4.92 (1.15)	5.02 (1.15)	*p* ≤ .001
	34.4%	24.2%	28.9%	*d* = −0.193
Decision	5.10 (1.10)	4.95 (1.09)	5.02 (1.09)	*p* ≤ .001
	33.4%	24.3%	28.5%	*d* = −0.137
Proactivity	4.78 (1.32)	4.77 (1.29)	4.77 (1.30)	*p* = .431
	25.4%	24.4%	24.9%	*d* = −.007
Perseverance	4.96 (1.27)	4.76 (1.27)	4.85 (1.27)	*p* ≤ .001
	31.1%	24.2%	27.4%	*d* = −0.160
Self‐assertion	5.00 (1.24)	4.66 (1.29)	4.82 (1.28)	*p* ≤ .001
	32.7%	21.5%	26.7%	*d* = −0.270
Contact	4.87 (1.33)	4.97 (1.28)	4.93 (1.31)	*p* = .044
	28.5%	30.3%	29.5%	*d* = 0.080
Group	4.82 (1.24)	4.93 (1.18)	4.88 (1.21)	*p* = .025
	24.6%	25.5%	25.1%	*d* = 0.089
Dyadic relations	4.81 (1.25)	5.00 (1.22)	4.91 (1.24)	*p* ≤ .001
	24.0%	29.1%	26.7%	*d* = 0.151
Self‐care	5.00 (1.26)	5.13 (1.20)	5.07 (1.23)	*p* = .007
	30.8%	33.6%	32.3%	*d* = 0.098
Mobility	5.29 (1.20)	5.17 (1.25)	5.22 (1.23)	*p* = .010
	40.2%	34.5%	37.2%	*d* = −0.092
Mini‐ICF‐APP mean score (global capacity level)	4.97 (0.90)	4.94 (0.85)	4.95 (0.87)	*p* = .307
				*d* = −0.041

*Note*: Each capacity dimension is rated from *7 = This is clearly a strength of mine* to *0* = *I am fully unfit to do this*. Means (standard deviation) are reported and percentage of participants who say that this capacity is clearly a strength of them or that they are better than most others in doing this (rating 6–7 on the respective capacity).

Although there are no significant differences on the global capacity level (i.e., in the Mini‐ICF‐APP‐S mean score), the best capacities differ between men and women: Men report better capacity levels than women in decision making, competency, perseverance, and assertiveness (Table 3), whereas women's self‐rating is stronger in dyadic relations, and self‐care (Table 3).

**FIGURE 1:**
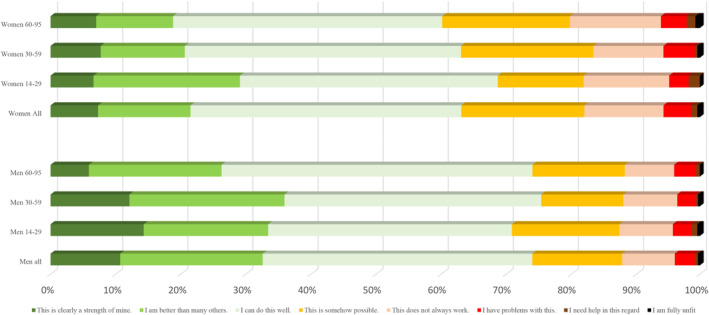
Assertiveness in men and women of different age groups in comparison.

**FIGURE 2:**
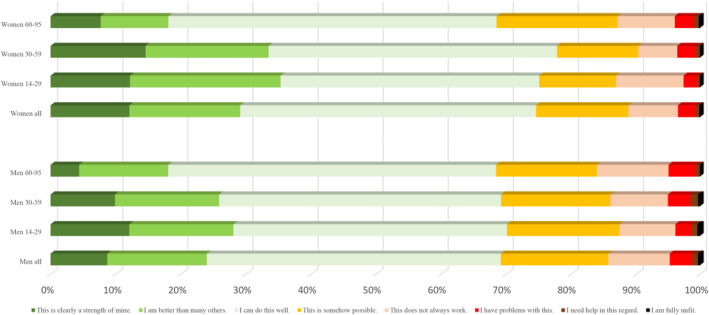
Capacity for dyadic relationships in men and women of different age groups in comparison.

**FIGURE 3:**
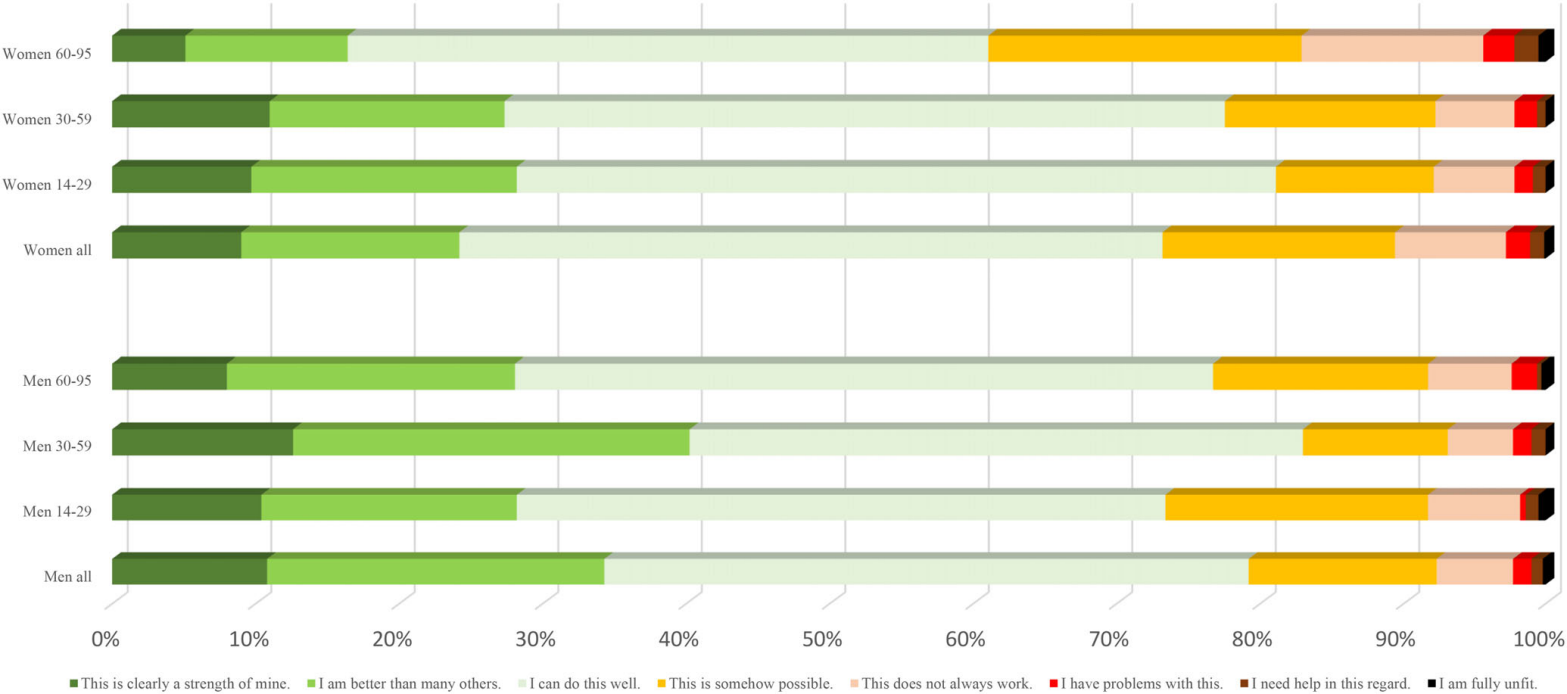
Capacity for applying competency and knowledge in men and women of different age groups in comparison.

**FIGURE 4:**
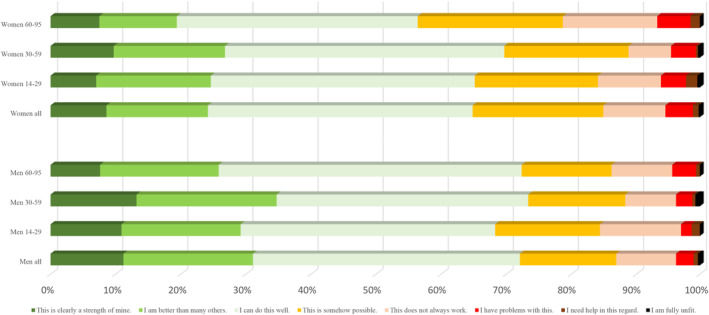
Perseverance and endurance in men and women of different age groups in comparison.

Regarding the capacities over the life span in more detail (Figures 1, 2, 3, 4), there are no significant differences between men and women in the age group 14–19 years. However, specific differences become observable up from the twenties and then seem to be stable until old age (70–95).

1. Assertiveness: Whereas men report best self‐assertion in their 30–59 years of life, there is an opposite tendency in women: in their mid‐age, women perceive themselves as less self‐asserted than in their twenties (Figure 1).
2. Dyadic relationships: Women in age groups 14–59 are more positive about their dyadic relationship capacities than men of the same age groups (Figure 2). Most women tend to perceive their dyadic relationship capacities strong or at least “well” in their 30–59 years. In older decades (60–95 years of age) both men and women perceive their dyadic relationship capacity on the same level.
3. Competence: Men of age groups 30–59 perceive their competences on the strongest level, as compared with the other male age groups: Nearly 40% say they are better than others, or competence is clearly a strength for them (Figure 3). In women there is no such difference of competence perception between the age groups: aged 14–59, women see their competences relatively stable (at least as “well”), but fewer than 30% say that this is clearly a strength for them, or they might be better as most others.
4. Endurance: In both women and men, endurance is perceived strongest in the 30–59 years old (in comparison with dyadic relationship which seem predominant in the age group of 14–29, Figure 4). Men perceive their endurance partly stronger, also in higher age groups (60–95).

## DISCUSSION

People from all age groups value their psychological capacities positively, about 50% say that at least one capacity is clearly a strength of theirs, or they are better than most others. This finding of rather (over‐)positive evaluation of one's own capacities and achievements is a typical finding in psychological research on traits and abilities. It is known as the better‐than‐average‐effect (Zell et al., 2020): General population usually think they are a bit better than most others. Specific results according to the research questions on psychological capacities in age groups and gender are discussed in the following.

### Capacity profiles in different age groups

The data show that different capacities are predominant in different life decades. Whereas capacities associated with dynamic, speed, situative action, and changes (proactivity, flexibility, small talk contacts, mobility) are perceived as strong in youth; capacities which require learning experiences, competencies and knowledge, are present in later decades of life. The reason can be that human learning development needs time until it reaches the state of a somewhat down‐to‐earth personality, being more competent and foresighted than in youth. This is a result of learning from life experiences and models. In conclusion, learning‐based capacities (i.e., how to apply competence, make decisions, assert oneself, keep dyadic relationships, and follow aims with endurance) can be distinguished from capacities which are rather based on innate drive or cognitive speed which result in proactivity behavior, flexibility, and mobility especially in younger years.

These findings support the idea of general cognitive development and intelligence development over the life span: Fluid intelligence capacities are strong in the two early life decades and then decrease (depending on weak or strong genetic constitution), whereas crystalline intelligence becomes stronger and more stable over the course of the life span even until older age (Cattell, 1987). Crystalline intelligence, namely, competency and knowledge, may compensate decreases in cognitive speed and fluency by selective optimization and compensation (Baltes & Baltes, 1990). With regard to the growing psychological demands and complexity of our modern working world, such capacity compensation becomes even more important for work performance (Mauno et al., 2020). Similar to cognitive intelligence, the model of psychological capacities can represent different peaks, and phases of increase, decrease, and (midlife) stability for different capacities.

### Capacity level and sociodemographic characteristics

The finding that higher capacity level is associated with better economic situation (here: fewer unemployment phases, higher income) is similar to other research that found cognitive achievements associated with socioeconomic success over the life span (Strenze, 2007).

It has also been found before that there were no systematic associations between gender and overall level of capacities (Feingold, 1988). The results from our representative study support this finding.

### Capacity differences in men and women

Perceived overall capacity level is not higher or lower in men or women. However, men and women report partly different levels of specific capacities: Men are more convinced about their capacities of competence and assertiveness, whereas women tend to value their dyadic relationship capacities stronger. This fits with empirical findings on gender differences in general cognitive achievements and in specific skills and activities. Concerning the overall cognitive achievements, there are no differences between women and men (except mathematics; Feingold, 1988). Gender differences become rather visible regarding specific skills. Young men were more likely to take a risk in investment decisions than young women (Booth & Katic, 2013). On the other hand, women were more likely to use interactive styles of management, and find coaching and developing effective (Burke & Collins, 2001). Coaching and developing employees requires trustful dyadic interaction with the coaches and thus requires dyadic relationship capacities in the leader. Similarly, women in our study valued their dyadic capacities better than did men of the same age groups.

In our study a greater percentage of men (as compared with women) was strongly convinced about their competence. Correspondingly, research on the classical cognitive abilities found that men give higher self‐estimates than women in most of cognitive abilities, except in verbal abilities where women see their strength (Syzmanowicz & Furnham, 2011).

### Limitations

Capacity levels and profiles as assessed in this representative study are self‐ratings. It cannot be concluded how persons would apply their psychological capacities in real life and real‐work context. There is no standard norm or anchor for self‐ratings of capacity levels. Participants give their ratings according to their own understanding of their life conditions and requirements. Thus, their capacity self‐perception has to be understood as a global attribution of satisfaction with their own skills. However, although individual anchors and life conditions may influence the ratings, self‐ratings are, nevertheless, of value and validity: The capacity self‐ratings are normally distributed, similar to personality traits. Gender and age group differences show that persons are able to differentiate in type and degrees of their capacities.

## CONCLUSION

This study provides, for the first time, prepresentative data on a broad range of work‐relevant cognitive, social, and basic psychological capacities (i.e., soft skills) according to an internationally validated ICF‐based capacity concept. The Mini‐ICF‐APP capacity concept refers to an internationally consented (mental) health understanding (ICF, WHO, 2001).

The study continues work‐related skills research, replicates earlier findings, and presents, for the first time, representative data on the broad set of psychologically relevant cognitive and noncognitive skills which are needed in the 21st century. These capacities are needed in general and work life (Geisinger, 2016; OECD, 2019). The concept of capacities according to the Mini‐ICF‐APP can be used in education, professional groups, vocational training, vocational reintegration and rehabilitation (Linden et al, 2009; AWMF, 2019). The data are useful for other studies for comparative purposes, and for estimating capacity distribution in specific employee age groups and gender.

## AUTHOR CONTRIBUTIONS

The author analyzed the data and wrote the manuscript. Data were collected by a professional representative survey institute USUMA GmbH https://www.usuma.com/.

## FUNDING INFORMATION

This research has been financially supported by the German Pension Fund. 0421/40‐64‐50‐01. The funder had no involvement in the study, design, or in the data collection and analysis.

## CONFLICT OF INTEREST STATEMENT

The author declares that she has no conflicts of interest.

## ETHICS STATEMENT

The study was reviewed and approved by the ethics committee of the Technische Universität Braunschweig (D‐2019‐03). Participants gave written informed consent to participate in the study before taking part.

## Data Availability

Data are available from the author upon reasonable request.
